# Formative feedback from the first-person perspective using Google Glass in a family medicine objective structured clinical examination station in the United States

**DOI:** 10.3352/jeehp.2018.15.5

**Published:** 2018-03-07

**Authors:** Julie Youm, Warren Wiechmann

**Affiliations:** Department of Emergency Medicine, University of California, Irvine School of Medicine, Orange, CA USA; Hallym University, Korea

**Keywords:** Google Glass, Formative feedback, Medical education, Communication, United States

## Abstract

**Purpose:**

This case study explored the use of Google Glass in a clinical examination scenario to capture the first-person perspective of a standardized patient as a way to provide formative feedback on students’ communication and empathy skills ‘through the patient’s eyes.’

**Methods:**

During a 3-year period between 2014 and 2017, third-year students enrolled in a family medicine clerkship participated in a Google Glass station during a summative clinical examination. At this station, standardized patients wore Google Glass to record an encounter focused on communication and empathy skills ‘through the patient’s eyes.’ Students completed an online survey using a 4-point Likert scale about their perspectives on Google Glass as a feedback tool (N= 255).

**Results:**

We found that the students’ experiences with Google Glass ‘through the patient’s eyes’ were largely positive and that students felt the feedback provided by the Google Glass recording to be helpful. Although a third of the students felt that Google Glass was a distraction, the majority believed that the first-person perspective recordings provided an opportunity for feedback that did not exist before.

**Conclusion:**

Continuing exploration of first-person perspective recordings using Google Glass to improve education on communication and empathy skills is warranted.

## Introduction

During the product announcement for Google Glass (GG) at the 2012 Google I/O conference, a team of skydivers flying above San Francisco used the device to livestream their jump to co-founder Sergey Brin, who was standing on the stage at the Moscone Center [1]. This dramatic introduction of a wearable computer with an optical head-mounted display inspired attention to a new form factor for mobile computing. GG displays information in a smartphonelike hands-free format that can respond to both voice commands and touch. It contains an on-board processor running the Android operating system, memory, display, WiFi, Bluetooth, and a camera with high-definition video capabilities. While GG itself cannot make phone calls, it can pair to an Android or iOS phone for calls and messaging. GG also comes equipped with some basic productivity apps including messaging, web browsing, and maps/directions.

GG is worn like a pair of glasses, making it an ideal device to support augmented reality and improving the degree to which the first-person perspective (1PP) can be shown by enabling photos and video to be captured from the perspective of the device wearer. These abilities of GG have led to much speculation about their potential application in areas such as the military, education, manufacturing, and healthcare. Pilot studies of GG in medical education have been conducted for procedural skills assessment [2], end-of-life training [3] and surgical education [4-6]. Our first initiatives using GG for medical education at the University of California, Irvine School of Medicine (UC Irvine) were in the objective structured clinical examination (OSCE). The OSCE is often used as a high-stakes summative evaluation of students’ clinical skills. For a 3-year period between 2014 and 2017, GG was integrated into the family medicine (FM) clerkship OSCE to explore the possibility of providing formative feedback to students based on a 1PP recording made using GG.

## Case presentation

### Ethical statement

This research was approved by the UC Irvine Institutional Review Board for Human Subjects (HS#2015-1781). Informed consent was received from each participant.

### Case

**Fig. 1.**
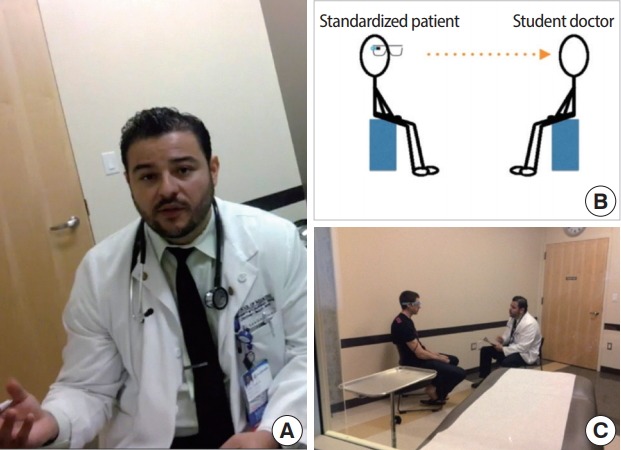
(A–C) ‘Through the patient’s eyes’ in the family medicine clerkship objective structured clinical examination. Written consent received from the student whos face appears above. A sample of a Google Glass video recording can be found in Supplement 1.

A critical component of the OSCE is an encounter between a student doctor and a standardized patient (SP). SPs are actors who simulate the symptoms of real patients and deliver standardized responses based on well-developed scripts. During an OSCE, students are recorded by fixed cameras in the examination rooms and are encouraged to review this recorded content afterwards to identify areas of improvement and further study. While the recordings are of acceptable quality, they are often captured from a ‘birds-eye’ perspective, limiting the depth of self-assessment by the students to gross deficiencies. Placing GG on the SP and recording the encounter creates a ‘through the patient’s eyes’ view (Fig. 1; written consent was received from the student whose face appears) that provides richer data about micro-expressions, body language, and tone of voice, and allows for the confirmation of proper physical exam technique (if applicable). Our hypothesis was that GG would help promote students’ reflective capacity, an important aspect of professional identity formation for humanistic physicians [7], by enabling a review of one’s own 1PP as part of formative feedback.

A scenario focused on assessing the communication and empathy skills of a student doctor was selected for GG integration into the FM OSCE. Every student who completed this OSCE participated in this experience. At the GG station, which had a total duration of 30 minutes, the SP started a GG recording prior to the start of the encounter and captured the SP’s 1PP of the student doctor during the entire encounter. After the student left the examination room, the SP stopped the GG recording, and handed the GG to the technical support team who downloaded the recorded video onto a MacBook using the ImageCapture app. Meanwhile, the SP immediately completed a checklist based on direct observations of the student. This ensured that the review and feedback of the GG recording did not have an impact on grading at the station. The downloaded videos were set to begin playback at an interaction surrounding a challenging question presented by the SP as a cue during the encounter. Students were invited back into the examination room, watched the cued videos for several minutes, and then received feedback on their communication skills from the SP during a 10-minute post-encounter session. Finally, students completed an online survey rating their experience and perceptions of GG as a feedback tool on a 4-point Likert scale after watching the recording and receiving feedback from the SP (N= 255). This survey was completed immediately at a designated computer after the GG station. The content validity of the GG survey was checked by the 2 authors through a process of reviewing results from a pilot study and discussion to reach 100% mutual agreement on the relevance of each question to the hypothesis. Responses to 4 of the negatively worded survey items (items #2, 3, 4, and 5 in Table 1) were reverse-coded so that a high value indicated the same type of response for all items to calculate the reliability of the survey using the Cronbach alpha. The Cronbach alpha for the 9 survey items was 0.80. The raw data are available in Supplement 2.

An analysis of the surveys about the integration of GG into the FM OSCE suggested that students found the feedback from the 1PP of the SP to be a positive experience (Table 1). Students (N= 255) agreed that the feedback from GG recordings was helpful (89%), that the recordings allowed an opportunity for feedback that did not previously exist (82%), and that they saw the value of GG in medical education (89%). Students also agreed that they were comfortable with the SP wearing GG during the encounter (84%) and that it did not affect their ability to communicate effectively with the SP (79%). Only 15% felt that GG negatively affected their performance during the encounter.

Ten percent of students encountered technical difficulties during the GG session. These technical difficulties included overheating of the GG causing a loss of the recording, poor audio quality, and the recording being accidentally stopped, either by the SP touching the GG or by voice commands from the SP or student (an option in later versions of the GG software). The line-of-sight limitations frequently cited for GG (e.g., in surgical settings [8]) were minimized in this scenario focused on communication skills where the 1PP is typically looking straight ahead.

## Discussion

We set out to test the feasibility of GG to capture first-person video from the SP perspective to provide feedback on communication and empathy skills. We found that students’ experience with GG during the FM OSCE was largely positive, and students felt that the feedback provided by the GG recording was helpful. More importantly, they believed that the 1PP recordings allowed an opportunity for feedback that did not exist previously, which supported our hypothesis that GG would help promote students’ reflective capacity. The GG recording was the first time that many students had an opportunity to see themselves from the patient’s perspective and reflect on their communication and empathy skills. For example, one student noticed, “I fiddle with my pen a lot!” while another realized, “some of my body movements I made weren’t ideal.”

It should be noted that 33% of students felt that GG was a distraction during the encounter. One student reported: “The bright light was a pretty big distraction and I could see myself being recorded in the glass.” There was also a shared concern from the students that this novel GG experience was conducted during a high-stakes OSCE and several stated that they would have preferred to receive this feedback in less formal settings earlier in medical school. However, as suggested by the survey results, students found this to be a positive experience in which they received useful and unique formative feedback despite potential distractions or technical limitations. “I noted that GG can at times break eye contact. That can be distracting; however, the feedback outweighs this problem.”

Overall, this case study suggests that GG should be further explored as a novel and effective feedback tool for use in medical education to provide formative feedback ‘through the patient’s eyes.’ The ability to provide feedback and instruction for point-of-care encounters in a practically feasible manner was a challenge prior to the emergence of this new wearable technology. Continuing exploration of this modality to improve education on communication and empathy skills is warranted, especially via the implementation of wearable technologies for students who seek innovative methods for empathy training [9]. The benefit of viewing oneself through the patient’s eyes can be summed up by the following student comment: “I enjoyed seeing myself in person speaking with the patient. I’ve never watched myself speaking with a patient before so I learned a lot about my speech, facial expressions, and overall tone with the patient. Knowing this will be useful for how I choose to interact with patients in the future.”

## Figures and Tables

**Table 1.** Survey on the use of Google Glass in a family medicine clerkship objective structured clinical examination (N=255)

Statement	Strongly disagree	Disagree	Agree	Strongly agree	Agreement^b)^ (%)	Mean ± standard deviation
1. I was comfortable with the SP wearing Google Glass during our encounter.	6	35	139	75	83.9	2.87 ± 0.72
2. Google Glass affected my ability to communicate effectively with the SP during our encounter.^a)^	58	143	47	7	21.2	2.01 ± 0.72
3. Google Glass was a distraction during our encounter.^a)^	54	118	76	7	32.5	2.14 ± 0.78
4. Knowing there was a Google Glass recording negatively affected my performance during this encounter.^a)^	60	157	34	4	14.9	1.93 ± 0.65
5. There were technical issues with Google Glass during our encounter.^a)^	181	49	19	6	9.8	1.41 ± 0.73
6. The feedback I received from the Google Glass recording was helpful.	5	24	139	87	88.6	3.21 ± 0.69
7. I feel that the Google Glass recording of me allowed an opportunity for feedback that did not exist.	9	37	120	89	82.0	3.13 ± 0.79
8. I look forward to more opportunities using Google Glass during my rotations.	11	62	131	51	71.4	2.87 ± 0.78
9. I see the value of using Google Glass for medical education.	7	21	154	73	89.0	3.15 ± 0.68

SP, standardized patient.

a) Item was *reverse-coded to calculate the* Cronbach’s alpha.

b) Agreement is represented by responses of “agree” or “strongly agree” on a 4-point scale.

## Supplementary materials

Supplement 1. Sample of Google Glass recording ‘Through the patient’s eyes’

Supplement 2. Data files are available from https://doi.org/10.7910/DVN/PWOY48

Supplement 3. Audio recording of the abstract
